# Surveillance of Zoonotic Pathogens and Taxonomic Identification of Non-volant Small Mammals in Peninsular Malaysia

**DOI:** 10.21315/tlsr2026.37.1.14

**Published:** 2026-03-31

**Authors:** Zhen Yun Siew, Nazifah Fitriyah Zariman, Wan Siti Mariam Wan Sa’idi, Zi Yi Lui, Harriydra Sai Muthu Coomarhesan, Isaac Seow, Nur Juliani Shafie, Siew Tung Wong, Mohd Firdaus Ariff Abdul Razak, Millawati Gani, Syriswin Wesdy Sindang, Kenny Voon

**Affiliations:** 1 Division of Biomedical Sciences, School of Pharmacy, University of Nottingham Malaysia, 43500 Semenyih, Selangor, Malaysia; 2 Department of Biological Sciences and Biotechnology, Faculty of Science and Technology, Universiti Kebangsaan Malaysia, 43600 Bangi, Selangor, Malaysia; 3 Faculty of Science and Marine Environment, Universiti Malaysia Terengganu, 21030 Kuala Terengganu, Terengganu, Malaysia; 4 Department of Pathology and Pharmacology, School of Medicine, IMU University, Bukit Jalil, 57000 Kuala Lumpur, Malaysia; 5 Ex-Situ Conservation Division, Department of Wildlife and National Parks (PERHILITAN), KM 10 Jalan Cheras, 56100 Kuala Lumpur, Malaysia; 6 Wildlife Conservation Division, Department of Wildlife and National Parks (PERHILITAN), KM 10 Jalan Cheras, 56100 Kuala Lumpur, Malaysia

**Keywords:** DNA Barcoding, Non-Invasive Sampling, Non-Volant Mammal, Zoonotic, Sanger Sequencing, DNA Barkod, Pensampelan Tidak Invasif, Mamalia Tidak Terbang, Zoonotik, Penjujukan Sanger

## Abstract

Malaysia’s tropical rainforests host a rich biodiversity, including various non-volant small mammals. Among these, murid rodents (family Muridae) are ecologically significant and frequently associated with zoonotic pathogens, making them important subjects for public health research. In recent years, treeshrews (family Tupaiidae), small omnivorous mammals once grouped with primates, have also gained increasing scientific attention due to their unique evolutionary position and emerging role in disease ecology. Rapid species identification is vital for effective surveillance, particularly in the context of emerging infectious diseases. In this study, PCR amplification targeting mitochondrial and nuclear DNA regions was performed using a range of primers, followed by Sanger sequencing to validate the amplicons. Among the primers tested, mcb398 and mcb869, targeting the mitochondrial cytochrome b gene, proved most effective, yielding consistent amplification and high-quality sequences for both rodents and treeshrews. Besides, 22 animals were captured and screened for selected zoonotic pathogens. *Paramyxoviruses, coronaviruses, picornaviruses, orthoreoviruses* and *Dengue viruses* were not detected in the faecal samples of rats, Asian house shrews and palm civets. However, *mammalian orthoreovirus* type 3 and *Dengue virus* serotype 2 were detected in one and three faecal samples from treeshrews, respectively. Notably, *Tupaia* sp. m ZYS-2025, detected in this study, may represent a novel species that has not known to science previously.

## Abstrak

Hutan hujan tropika di Malaysia mempunyai kepelbagaian dan kelimpahan biologi yang tinggi, termasuklah pelbagai mamalia kecil tidak terbang. Antara kumpulan mamalia kecil ini, tikus murid (famili Muridae) memainkan peranan yang penting dari segi ekologi. Tikus murid juga sering dikaitkan dengan patogen zoonotik dan menjadikannya subjek utama dalam penyelidikan berkaitan kesihatan awam. Dalam beberapa tahun kebelakangan ini, tupai tanah (famili Tupaiidae), iaitu mamalia kecil omnivor yang dahulunya dikelaskan bersama primat, turut mendapat perhatian saintifik. Hal ini disebabkan oleh kedudukan evolusi tupai tanah yang unik serta peranan mereka yang semakin dikenali di dalam ekologi penyakit. Proses pengecaman spesies yang pantas adalah amat penting untuk tujuan pemantauan, terutamanya dalam konteks kemunculan penyakit berjangkit yang semakin membimbangkan. Dalam kajian ini, amplifikasi PCR yang menyasarkan rantau DNA mitokondria dan nuklear telah dijalankan dengan menggunakan pelbagai primer. Selepas itu, produk amplifikasi PCR ini telah disahkan dengan menggunakan penjujukan Sanger. Antara primer yang digunakan, set primer mcb398 dan mcb869 yang menyasarkan gen sitokrom b mitokondria telah menunjukkan keberkesanan yang paling tinggi. Set primer ini menghasilkan amplifikasi yang konsisten serta jujukan yang berkualiti tinggi untuk kedua-dua tikus dan tupai tanah. Pada masa yang sama, 22 haiwan telah ditangkap dan disaring untuk mengenal pasti kewujudan patogen zoonotik terpilih. Paramyxoviruses, coronaviruses, picornaviruses, orthoreoviruses dan Dengue virus tidak dikesan di dalam semua sampel najis tikus, cencurut dan musang pulut. Walau bagaimanapun, mammalian orthoreovirus serotaip 3 dikesan di dalam satu sampel najis tupai tanah. Manakala, Dengue virus serotaip 2 dikesan dalam tiga sampel najis tupai tanah. Menariknya, Tupaia sp. m ZYS-2025 yang dijumpai dalam kajian ini mungkin mewakili spesies baharu yang belum dilaporkan dalam sains sebelum ini.

**HIGHLIGHTS**

- At least five non-volant small mammals were identified in Semenyih.
- Screening of animal faeces revealed no detectable zoonotic viruses.
- *Tupaia* sp. m ZYS-2025 may represent a novel *Tupaia* species.

## INTRODUCTION

Malaysia is recognised for its highly diverse tropical rainforests, which support exceptional biodiversity, with over 360 mammal species documented. Among these, non-volant small mammals are particularly significant due to their essential ecological roles, including seed dispersal, nutrient cycling and serving as both prey and predators (Munian *et al*. 2020). Besides, these small mammals also serve as important reservoirs for numerous microorganisms, primarily due to their diverse interactions with a range of habitats and species. Among these mammals, rodents, particularly those from the family *Muridae*, play key ecological roles and are frequently associated with zoonotic pathogens, making them a focal point of public health research. Notably, one rat species from the genus *Niviventer* has been classified as Endangered, and another as Vulnerable, according to the Red List of Mammals for Peninsular Malaysia Version 2.0 (PERHILITAN 2017).

Besides rodents, treeshrews (Order: Scandentia), another group of small non-volant mammals, have garnered growing scientific interest due to their ecological significance, adaptability and potential role as reservoir hosts for various zoonotic pathogens that pose a threat to humans. These mammals are widely distributed across a range of habitats, including primary forests, secondary growth areas, suburban environments and even urban settings, demonstrating their ecological flexibility (Ab Hamid *et al*. 2025; Siew *et al*. 2024). Recent studies have highlighted their role in pathogen ecology, as they have been found to harbour a diverse array of zoonotic and vector-borne agents. Some pathogens commonly detected in treeshrews include *Mycobacterium* sp., *Orientia tsutsugamushi*, *Borrelia* sp., filarial parasites, *Leptospira* sp. and many others (Mohd-Taib *et al*. 2020; Mohd-Azami *et al*. 2023; Mat Udin *et al*. 2020; Siew *et al*. 2024; Siew *et al*. 2025a). The presence of these pathogens suggests that treeshrews may contribute to the maintenance and transmission of infectious agents in the environment, particularly in areas where human-wildlife interfaces are expanding. As such, treeshrews represent an important yet understudied component of wildlife surveillance programs aimed at understanding emerging infectious disease risks. The authors would like to emphasise that many other viruses and hosts of interest, particularly arboviruses responsible for tropical outbreaks (Siew *et al*. 2025c), exist. However, not all were covered in this study.

Accurate, sustainable, affordable, convenient and rapid species identification is crucial for effective monitoring and surveillance of small mammal populations, especially in the context of emerging infectious diseases. It also plays a key role in biodiversity conservation and ecological studies. In this study, we assessed the performance of several primer sets targeting mitochondrial and nuclear DNA regions to evaluate their effectiveness in identifying common small mammal species, with a focus on *Rattus* sp. and *Tupaia* sp. only. In parallel, molecular screening for selected zoonotic viruses was performed using PCR-based detection methods to determine the presence and prevalence of each virus.

## MATERIALS AND METHODS

This project was reviewed and approved by the Department of Wildlife and National Parks (PERHILITAN), Peninsular Malaysia (Reference Nos.: JPHLTN.600-6/1/4 JLD5 (79) and JPHLTN.600-6/1/4 JLD2 ()), as well as the Northern Terengganu District Forestry Office (Ref. No.: PHDTU.1/5/15 BHG.12) under the Ministry of Natural Resources and Environmental Sustainability. Ethical clearance was also obtained from the Animal Welfare and Ethical Review Body (AWERB) and Universiti Malaysia Terengganu (Ref. No.: UMT/JKEPHMK/2023/83).

A “landmine” trapping approach was developed to enhance the efficiency of animal capture and sample collection. In this method, biodegradable teabags filled with cat food were placed around the perimeter of the plantation area, spaced approximately 10 m–20 m apart. The teabags were checked daily, and any that were found destroyed with the bait consumed were promptly replaced. A cage trap baited with cat food was then deployed at any location where bait consumption was observed on at least two separate occasions. Cage traps were set only after 7:00 PM and inspected daily at approximately 6:00 AM. Upon capture, animal faeces and urine were immediately collected using sterile wooden swabs and transferred into viral transport medium (VTM) contained in either 15 mL or 50 mL tubes, while fur samples were preserved in 1.5 mL tubes containing absolute ethanol. A photograph of each captured animal was also taken for documentation purposes. Only non-invasive sampling was conducted on animals captured at the University of Nottingham Malaysia while treeshrew organ samples were provided by Universiti Malaysia Terengganu (UMT).

For small mammal identification, the 1.5 mL tubes with animal fur were left in a biosafety cabinet (BSC) or laminar airflow (LAF) to evaporate A ethanol. Then, total nucleic acid extraction was performed using the PrimeWay Viral DNA/RNA Extraction Kit (1st BASE, Malaysia), strictly following the manufacturer’s protocol. The extracted DNA was utilised in the polymerase chain reaction (PCR) amplification using the exTEN II PCR Master Mix (1st BASE, Malaysia).

A priori sample size calculation was not performed due to field and logistical constraints inherent to wildlife trapping. Therefore, a post hoc sample size estimation was conducted based on prevalence estimation. Using a 95% confidence level and assuming an expected prevalence of 5%, the minimum required sample size was estimated using the formula:


$$n=\frac{Z^{2}\times P\times (1-P)}{d^{2}}\times 100$$

Where *n* is the required sample size, *Z* is the Z-score for the confidence level, *P* isthe expected or observed prevalence, and *d* is the desired precision or the margin of error. The obtained sample size in this study meets the requirement for estimating a 5% prevalence with ± 10% precision, and provides preliminary but meaningful insights into the presence and prevalence of the targeted zoonotic viruses.

For virus detection, samples preserved in VTM were vortexed until homogenised and centrifuged at 4,000 rpm for 5 min. Subsequently, 1.5 mL of the supernatant was transferred into a 2 mL microcentrifuge tube and further centrifuged at a minimum of 10,000 rpm for 20 min. The resulting supernatant was then used for total nucleic acid extraction as described above. Then, cDNA synthesis was performed using the ReverTra Ace qPCR RT Master Mix with gDNA Remover (Toyobo, Japan). For double-stranded RNA (dsRNA) virus targets, an additional denaturation step was performed by incubating the RNA at 95°C for 1 min, followed by immediate chilling on ice before proceeding with cDNA synthesis. The resulting cDNA was subsequently used for PCR amplification, as described above, using different sets of primers.

The general PCR thermal profile consisted of an initial denaturation at 95°C for 2 min, followed by 35 cycles of denaturation at 95°C for 40 sec, annealing at 50°C–58°C for 40 sec, and extension at 72°C for 1 min per kilobase (kb). A final extension was carried out at 72°C for 5 min for amplicons < 1 kb, or 7 min for amplicons > 1 kb.

Following PCR amplification, a 2% agarose gel was prepared using biotechnology-grade agarose powder (1st BASE, Malaysia) and 10X Tris-Borate-EDTA (TBE) buffer, pH 8.3, ultra-pure grade (1st BASE, Malaysia). Subsequently, 5 μL of PCR product and a 100 bp Plus DNA Ladder (MK004-2; Hefei Bomei Biotechnology, China) were loaded into each well. Gel electrophoresis was performed at 90 V for 50 min.

In total, 6 sets and 15 sets of primers were adopted from other studies for small mammal identification and virus detection (Supplementary Material Table S1), respectively. All primers were synthesised by Integrated DNA Technologies (IDT), Singapore.

Finally, all amplified PCR products with the correct amplicon size were sent for Sanger sequencing as demonstrated previously (Siew *et al*. 2023; 2024). Briefly, the DNA band obtained from gel electrophoresis was extracted using the PrimeWay Gel Extraction/PCR Purification Kit (1st BASE, Malaysia). Then, the purified PCR products were subjected to bidirectional sequencing with their respective forward and reverse primers using BigDye® Terminator version 3.1 Cycle Sequencing Kit (Thermo Fisher Scientific, USA).

## RESULTS

**FIGURE 1:**
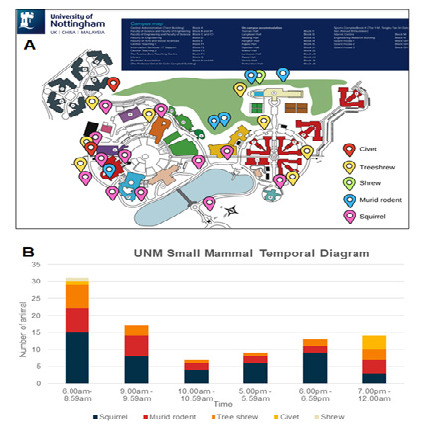
Spatial and temporal distribution diagram of non-volant small mammals observed in the University of Nottingham Malaysia. (A) Spatial diagram of non-volant small mammals observed. (B) Temporal diagram of non-volant small mammals observed.

From 2022 to 2025, at least five types of non-volant small mammals were observed at the University of Nottingham Malaysia. Frequently sighted species included treeshrews (*Tupaia* sp.), rats (*Rattus* sp.) and common squirrels (*Callosciurus* sp.). The palm civet (*Paradoxurus* sp.) was occasionally observed, particularly during the fruiting season of the Java apple, as reported by Siew *et al*. (2024). The Asian house shrew (*Suncus murinus*) was also recorded, though much less frequently. Notably, a civet resembling the Malayan civet (*Viverra tangalunga*) was observed only once in 2023. Spatial and temporal distribution diagrams of non-volant small mammals were generated based on observations recorded from January to May 2025 (Fig. 1).

**FIGURE 2:**
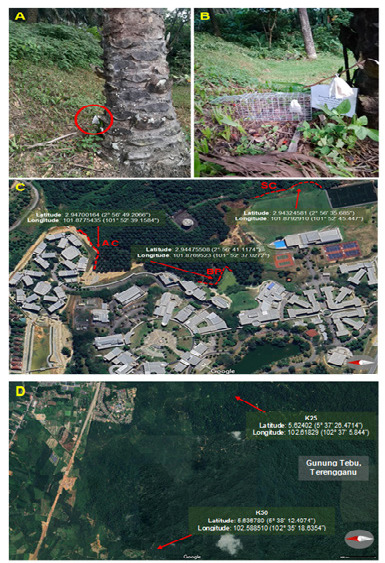
The “landmine” trapping layout at three different locations. (A) A teabag (circled in red) containing cat food was hung on an oil palm tree. (B) A cage trap was set up at a site where animal activity was suspected. (C) Location AC is near the accommodation area; Location BP is beside a small pond near the cafeteria; and Location SC is located behind the sports complex. (D) K25 and K50 are two trapping locations in Gunung Tebu, Terengganu.

The “landmine” trapping method using a teabag baited with cat food was successfully deployed (Fig. 2), with the first successful capture occurring on day 3 post-deployment. The trapping activities in Terengganu were also successful (Fig. 2). In total, 22 animals were captured at University of Nottingham Malaysia (Supplementary Material Fig. S1), and samples were collected. Additionally, organs from 12 treeshrews were provided by UMT.

Only the primer set mcb398/mcb869 successfully amplified DNA from all rats and treeshrews (Table 1 and Supplementary Material Fig. S2A). The primer sets MurND5 and 16S-3 were able to correctly amplify rat DNA, but either produced bands of incorrect amplicon size or sequences that were undefined for treeshrews. The primer set E18F/E1772R only amplified some rat samples, even after two repetitions. The primer set LCO1490/HCO2198 amplified DNA from all animal samples. However, usable results were only obtained from DNA extracted from internal organs. Amplification using fur samples resulted in the detection of fungal DNA, specifically *Malassezia japonica*, indicating contamination or overgrowth on the fur surface. All sequences obtained have been deposited in GenBank (Table 2).

None of the targeted viruses were detected in any of the captured animals, even after two independent rounds of PCR screening were performed (Supplementary Material Fig. S2B). However, *Mammalian orthoreovirus* serotype 3 was detected in one faecal sample from a University of Nottingham Malaysia treeshrew, and *Dengue virus* serotype 2 was detected in three other samples. Detailed data have been published elsewhere (Siew *et al*. 2025a; 2026).

## DISCUSSION

The University of Nottingham Malaysia campus is located at the border between Semenyih, Selangor, and Negeri Sembilan. The university compound is situated in a suburban area surrounded by oil palm plantations and forested patches, which provide habitats for a variety of wildlife. Among these, non-volant small mammals are frequently observed in and around the campus. Similarly, treeshrews have been observed in great numbers in Terengganu.

In this study, we confirmed the presence of at least five types of non-volant small mammals: treeshrews (*Tupaia* sp.), rats (*Rattus* spp.), common squirrels (*Callosciurus* sp.), palm civets (*Paradoxurus* sp.) and Asian house shrews (*Suncus murinus*). Notably, treeshrews and rats are often associated with zoonotic pathogens and are of particular public health interest (Mohd-Taib *et al*. 2020; Mohd-Azami *et al*. 2023; Mat Udin *et al*. 2020; Siew *et al*. 2024).

We identified three species of rats: *Rattus norvegicus*, *Rattus* sp. R3 and *Rattus tiomanicus*, with *R. norvegicus* being the most dominant, followed by *Rattus* sp. R3 and *R. tiomanicus*. Interestingly, discrepancies between mitochondrial DNA and nuclear 18S rRNA gene sequences were observed in rats R5, R6 and R11, suggesting possible genetic recombination or introgression events. For treeshrews, only one species, *Tupaia* sp. m ZYS-2025, was identified. Similarly, *Tupaia* sp. m ZYS-2025 showed close similarity to the common treeshrew (*Tupaia glis*) based on cytochrome c oxidase subunit I (COI) gene analysis. However, its cytochrome *b* gene shared less than 90% similarity with *Tupaia belangeri*, a rarely reported treeshrew species in Peninsular Malaysia. These findings suggest that *Tupaia* sp. m ZYS-2025 may represent a novel species first documented in this study. Unfortunately, the whole genome sequence and voucher specimens for this species have not yet been obtained.

As demonstrated in this study, the primer set mcb398/mcb869 was the most effective for identifying both rats and treeshrews. For rat fur samples, mcb398/mcb869 was followed in performance by MurND5, 16S-3, and E18F/E1772R. In contrast, for treeshrew samples, only the mcb398/mcb869 and LCO1490/HCO2198 primer sets produced reliable results. To optimise both cost and efficiency, primers can be evaluated using various online primer analysis tools (Table 3) and compared based on pricing (Table 4). For instance, the LCO1490/HCO2198 primer set is approximately five times cheaper than the redesigned jgLCO1490/jgHCO2198 primer set by Geller *et al*. (2013).

## CONCLUSION

Primers mcb398 and mcb869, which target the mitochondrial cytochrome *b* gene, were the most effective, producing consistent amplification and high-quality sequences for both rodents and treeshrews. No evidence of *paramyxoviruses*, *coronaviruses*, *picornaviruses*, *orthoreoviruses* or *Dengue virus* was found in the faecal samples of rats, Asian house shrews, or palm civets. However, *Mammalian orthoreovirus* type 3 and *Dengue virus* serotype 2 were detected in one and three faecal samples from treeshrews, respectively. *Tupaia* sp. m ZYS-2025 may represent a novel species first documented in this study.

## SUPPLEMENTARY MATERIALS

FIGURE S1 Photographs of captured animals and the non-invasive sampling method. (A) A large-sized brown rat (*Rattus norvegicus*). (B) A small-sized Malaysian field rat (*Rattus tiomanicus*). (C) An Asian house shrew (*Suncus murinus*) caught while attempting to escape from the cage. (D) All captured animals were covered with a biohazard bag and placed in a dark, undisturbed area. (E) A common palm cived (*Paradoxurus hermaphroditus*). (F) Faecal materials and urine of the palm civet were easily collected from the biohazard bag. (G) A treeshrew from Gunung Tebu, Terengganu.

FIGURE S2 Representative figure of gel electrophoresis results for (A) small mammal molecular identification and (B) virus detection. (A) The expected amplicon sizes were successfully obtained for all rat (R) samples, except for the cytochrome c oxidase subunit I (*COI*) gene, which was excluded due to non-specific amplification of a fungal gene. In treeshaw (T) samples, non-specific or failed amplification was observed for several primer sets, except for mcb398/mcb869 (mcb), 16S-3 and *COI*. However, subsequent Sanger sequencing revea;ed that the 16S-3 amplification was also non-specific. (B) No viral amplicons were detected in any of the assays. NTC = no template control. Lanes 1 to 15 correspond to the 15 primer sets listed in Table 2, ranging from PAR-F1/PAR-R (1) to Dcon-F/DENV4-R (15).

**TABLE S1:** Primer sequences for (A) DNA barcoding of non-volant small mammals and (B) virus dectection.

Target	Type of nucleic acid	Primer	Sequence (5′ – 3′)	Amplicon size (bp)	Reference
A. DNA barcoding of non-volant small mammals					

18S rRNA	Nuclear	E18F	GATCCMGGTTGATYCTGCC	~1740	( Hongoh & Toyoda 2011)
		E1772R	CWDCBGCAGGTTCACCTAC		

Cytochrome c oxidase subunit I	Mitochondrial	LCO1490	GGTCAACAAATCATAAAGATATTGG	658	( Folmer *et al*. 1994; Geller *et al*. 2013)
		HCO2198	TAAACTTCAGGGTGACCAAAAAATCA		
		
NADH dehydrogenase subunit 5		MurND5F	GCAGTTCTCTTCATGATAYATAC	919	( Masnaini *et al*. 2023)
		MurND5R	GTTTCAGGCGTTGGTGTT		
			
Cytochrome *b*		mcb398	TACCATGAGGACAAATATCATTCTG	472	( Verma & Singh 2002)
		mcb869	CCTCCTAGTTTGTTAGGGATTGATCG		
			
16S rRNA		16S-3F	AAGACGAGAAGACCCTATGGA	209~265	( Xue *et al*. 2017)
		16S-3R	GATTGCGCTGTTATCCCTAGGGTA		
		
mtDNA		mtDNAF	CCTCCCTAAGACTCAAGGAA	385~787	
		mtDNAR	CGGAGCGAGAAGAGG		

B. Virus dectection					

Paramyxoviridae	RNA	PAR-F1	GAAGGITATTGTCAIAARNTNTGGAC	200~500	( Tong *et al*. 2008)
		PAR-R	GCTGAAGTTACIGGITCICCDATRTTNC		
		
Respirovirus, Morbillivirus, Henipavirus		RES-MOR-HEN-F1	TCITTCTTTAGAACITTYGGNCAYCC		
		RES-MOR-HEN-R	CTCATTTTGTAIGTCATYTTNGCRAA		
		
Avulavirus, Rubulavirus		AVU-RUB-F1	GGTTATCCTCATTTITTYGARTGGATHCA		
		AVU-RUB-R	GCAATTGCTTGATTITCICCYTGNAC		
		
Pneumovirinae		PNE-F1	GTGTAGGTAGIATGTTYGCNATGCARCC		
		PNE-R	GTCCCACAAITTTTGRCACCANCCYTC		
		
Coronaviridae		Q-CoVF1	CGTTGGIACWAAYBTVCCWYTICARBTRGG	~520	( Quan *et al*. 2010)
		Q-CoVR1	GGTCATKATAGCRTCAVMASWWGCNACATG		
	
		X-CoVOutF1	CCAARTTYTAYGGHGGITGG	~670	( Xiu *et al*. 2020)
		X-CoVOutR1	TGTTGIGARCARAAYTCATGIGG		

Picornaviridae		RVF	GAAACACGGACACCCAAAGTA	130	( Blomqvist *et al*. 1999)
		RVR	TCCTCCGGCCCCTGAATG		
		
Human parainfluenza virus type 1 HN gene		PF526	ATTTCTGGAGATGTCCCGTAGGAGAAC	200	( Fan & Henrickson 1996)
		PR678	CACATCCTTGAGTGATTAAGTTTGATGA		
		
Pteropine orthoreovirus		PRVMiyazakiS4F2	CAACTTCCACTCGTTCGTTG	238	( Siew *et al*. 2025b)
		PRVMiyazakiS4R2	GATGATGTGGAAACGGATAC		
		
MRV L1 segment		MRV-L1F	TTCACTCAGGCATTATCCGA	560	( Mao *et al*. 2024)
		MRV-L1R	TCCGCTTCTGACTCCTGA		
		
MRV S1 segment		MRV-S1c	ATGGATCCTCGCTTACGTGA	~500	( Li *et al*. 2015)
		MRC-S1d	GCATCCATTGTAAATGACGAGTCTG		

Dengue virus		Dcon-F	AGTTGTTAGTCTACGTGGACCGACA		( Siew *et al*. 2023)
		DENV1-R	CGTCTCAGTGATCCGGGGG	613	
		DENV2-R	CGCCACAAGGGCCATGAACAG	252	
		DENV3-R	TAACATCATCATGAGACAGAGC	390	
		DENV4-R	CTCTGTTGTCTTAAACAAGAGA	492	

## Figures and Tables

**TABLE 1:** Species identification of the non-volant small mammals.

ID	mtDNA	MurND5	mcb398 & mcb869	16S-3	E18F & E1772R	LCO1490 & HCO2198
R1	-	*Rattus norvegicus*	*Rattus norvegicus*	*Rattus norvegicus*	-	-
R2	-	*Rattus norvegicus*	*Rattus norvegicus*	*Rattus norvegicus*	-	-
R3	-	Rattus norvegicus	Rattus norvegicus	*Rattus norvegicus*	*Rattus norvegicus*	-
R4	-	*Rattus norvegicus*	*Rattus norvegicus*	*Rattus norvegicus*	-	-
R5	-	*Rattus* sp. R3	*Rattus* sp. R3	*Rattus* sp. R3	*Rattus norvegicus*	-
R6	-	Rattus tiomanicus	*Rattus tiomanicus*	*Rattus tiomanicus*	*Rattus* sp. R3	-
R7	-	*Rattus* sp. R3	*Rattus* sp. R3	*Rattus* sp. R3	*Rattus* sp. R3	-
R8	-	*Rattus* sp. R3	*Rattus* sp. R3	*Rattus* sp. R3	*Rattus* sp. R3	-
R9	-	*Rattus norvegicus*	*Rattus norvegicus*	*Rattus norvegicus*	*Rattus norvegicus*	-
R10	-	*Rattus norvegicus*	*Rattus norvegicus*	*Rattus norvegicus*	*Rattus norvegicus*	-
R11	-	*Rattus tiomanicus*	*Rattus tiomanicus*	*Rattus tiomanicus*	*Rattus* sp. R3	-
R12	-	*Rattus norvegicus*	*Rattus norvegicus*	*Rattus norvegicus*	-	-
R13	-	*Rattus norvegicus*	*Rattus norvegicus*	*Rattus norvegicus*	*Rattus norvegicus*	-
R14	-	*Rattus norvegicus*	*Rattus norvegicus*	*Rattus norvegicus*	-	-
R15	-	*Rattus* sp. R3	*Rattus* sp. R3	*Rattus* sp. R3	*Rattus* sp. R3	-
R16	-	*Rattus norvegicus*	*Rattus norvegicus*	*Rattus norvegicus*	*Rattus norvegicus*	-
R17	-	*Rattus norvegicus*	*Rattus norvegicus*	*Rattus norvegicus*	-	-
T1	-	-	*Tupaia* sp. m ZYS-2025	-	-	*Tupaia* sp. m ZYS-2025
T2	-	-	*Tupaia* sp. m ZYS-2025	-	-	*Tupaia* sp. m ZYS-2025
T3	-	-	*Tupaia* sp. m ZYS-2025	-	-	*Tupaia* sp. m ZYS-2025
T4	-	-	*Tupaia* sp. m ZYS-2025	-	-	*Tupaia* sp. m ZYS-2025
T5	-	-	*Tupaia* sp. m ZYS-2025	-	-	*Tupaia* sp. m ZYS-2025
T6	-	-	*Tupaia* sp. m ZYS-2025	-	-	*Tupaia* sp. m ZYS-2025
T7	-	-	*Tupaia* sp. m ZYS-2025	-	-	*Tupaia* sp. m ZYS-2025
T8	-	-	*Tupaia* sp. m ZYS-2025	-	-	*Tupaia* sp. m ZYS-2025
T9	-	-	*Tupaia* sp. m ZYS-2025	-	-	*Tupaia* sp. m ZYS-2025
T10	-	-	*Tupaia* sp. m ZYS-2025	-	-	*Tupaia* sp. m ZYS-2025
T11	-	-	*Tupaia* sp. m ZYS-2025	-	-	*Tupaia* sp. m ZYS-2025
T12	-	-	*Tupaia* sp. m ZYS-2025	-	-	*Tupaia* sp. m ZYS-2025
T13	-	-	*Tupaia* sp. m ZYS-2025	-	-	-
T14	-	-	*Tupaia* sp. m ZYS-2025	-	-	-
T15	-	-	*Tupaia* sp. m ZYS-2025	-	-	-

*Note*: Molecular identification was not conducted for the Asian house shrew and the palm civet.

**TABLE 2:** GenBank accession numbers of all distinct sequences obtained.

Species	Primer set	GenBank accession number
*Rattus norvegicus*	MurND5	PV440193–PV440195
*Rattus* sp. R3		PV440196, PV440197
*Rattus tiomanicus*		PV440198

*Rattus norvegicus*	16S-3	PV383293–PV383295
*Rattus* sp. R3		PV383296, PV383297
*Rattus tiomanicus*		PV383298

*Rattus norvegicus*	E18F/E1772R	PV383289, PV383290, PV383292
*Rattus* sp. R3		PV383291
*Rattus tiomanicus*		-

*Rattus norvegicus*	mcb398/mcb869	PV440187–PV440189
*Rattus* sp. R3		PV440190, PV440191
*Rattus tiomanicus*		PV440192
*Tupaia* sp. m ZYS-2025		PV440199

*Tupaia* sp. m ZYS-2025	LCO1490/HCO2198	PV441709, PV441710
*Malassezia japonica*		PV440186

**TABLE 3:** Primer analysis using different primer analysis tools.

Primer	Tm (°C)^a^	CG (%)	Extinction coefficient (l/(mol·cm)	Molecular weight (g/mol)	nmol/OD260	μg/OD260	ΔG (kcal.mole^−1^)^b^	Primer-dimer estimation^c^
E18F	64.7	57.9	172550.0	5775.3	5.80	33.47	0.28	Negative
E1772R	61.0	57.9	172683.2	5733.3	5.79	33.21	−1.69	
LCO1490	60.6	32.0	263100.0	7722.1	3.80	29.35	−1.62	Negative
HCO2198	66.3	34.6	268200.0	7980.3	3.73	29.75	−2.33	
MurND5F	54.9	37.0	219850.0	6981.1	4.55	31.75	0.20	Negative
MurND5R	60.5	50.0	166300.0	5567.7	6.01	33.48	−0.11	
mcb398	62.0	36.0	250500.0	7649.1	3.99	30.54	−1.60	Negative
mcb869	66.6	46.2	244000.0	7983.2	4.10	32.72	−1.84	
16S-3F	60.9	47.6	223600.0	6497.3	4.47	29.06	−1.29	Cross primer dimers
16S-3R	67.0	50.0	228000.0	7374.8	4.39	32.35	−0.84	
mtDNAF	59.2	50.0	194400.0	6055.0	5.14	31.15	−1.31	Negative
mtDNAR	57.3	66.7	160100.0	4716.1	6.25	29.46	0.14	

*Notes*: a = The parameters for Tm calculation were set at a primer concentration of 1 μM and a salt concentration of 50 mM. The Tm was predicted using a modified nearest-neighbour method as described by Breslauer *et al*. (1986); b = Default qPCR parameter settings were used for target type and concentrations: DNA target, 1 μM oligonucleotide, 50 mM Na^+^, 3 mM Mg^2+^ and 0.8 mM dNTPs; c = The sensitivity setting for primer-dimer estimation was set to 3 (optimal). Analyses were performed using the Multiple Primer Analyzer (Thermo Fisher) and OligoAnalyzer (IDT). While many additional parameters and analyses are available on these platforms, they were not included in the table. Other available primer design and analysis tools include: Oligo Analysis Tool, PCR Primer Stats, PrimerQuest Tool, OligoEvaluator, NetPrimer and Primer3web. It should be noted that different tools may implement distinct algorithms for primer analysis, potentially resulting in slight variations in outcomes.

**TABLE 4:** List of primer prices.

Primer	Price (RM/100 nmol)^a^	Primer set price (RM/100 nmol)	Primer set price (USD/100 nmol)^b^
E18F	41.80	83.60	19.46
E1772R	41.80		
LCO1490	55.00	112.20	26.12
HCO2198	57.20		
jgLCO1490	276.60	555.30	129.26
jgHCO2198	278.70		
MurND5F	50.60	90.20	21.00
MurND5R	39.60		
mcb398	55.00	112.20	26.12
mcb869	57.20		
16S-3F	46.20	99.00	23.04
16S-3R	52.80		
mtDNAF	44.00	77.00	17.92
mtDNAR	33.00		

*Notes:* a = Primer prices were obtained from Apical Scientific Sdn. Bhd., the local distributor of Integrated DNA Technologies. Prices are valid until 8 June 2025; b = Currency conversion was based on an exchange rate of 1 USD = RM4.30 as of 16 May 2025.
