# Supplementary figures and images for: Surveillance of Zoonotic Pathogens and Taxonomic Identification of Non-volant Small Mammals in Peninsular Malaysia
Source: Trop Life Sci Res. 2026 Mar 31;37(1):293–313. doi: 10.21315/tlsr2026.37.1.14 (PMC13127999; doi:10.21315/tlsr2026.37.1.14)

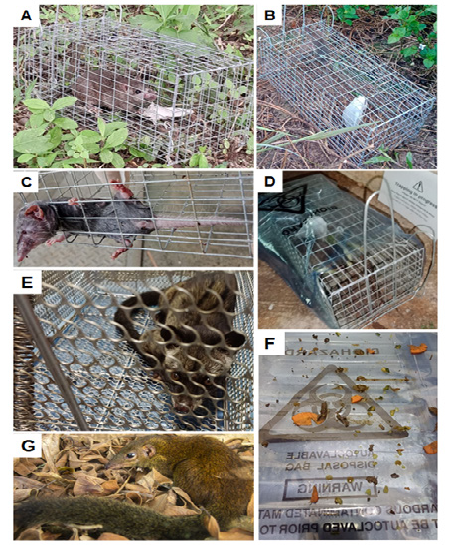

Supplement: FIGURE S1 — Photographs of captured animals and the non-invasive sampling method. (A) A large-sized brown rat (Rattus norvegicus). (B) A small-sized Malaysian field rat (Rattus tiomanicus). (C) An Asian house shrew (Suncus murinus) caught while attempting to escape from the cage. (D) All captured animals were covered with a biohazard bag and placed in a dark, undisturbed area. (E) A common palm cived (Paradoxurus hermaphroditus). (F) Faecal materials and urine of the palm civet were easily collected from the biohazard bag. (G) A treeshrew from Gunung Tebu, Terengganu. [file TLSR_37-1-293-g00S1.tif]

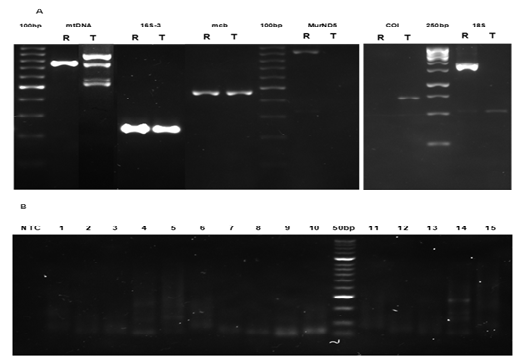

Supplement: FIGURE S2 — Representative figure of gel electrophoresis results for (A) small mammal molecular identification and (B) virus detection. (A) The expected amplicon sizes were successfully obtained for all rat (R) samples, except for the cytochrome c oxidase subunit I (COI) gene, which was excluded due to non-specific amplification of a fungal gene. In treeshaw (T) samples, non-specific or failed amplification was observed for several primer sets, except for mcb398/mcb869 (mcb), 16S-3 and COI. However, subsequent Sanger sequencing revea;ed that the 16S-3 amplification was also non-specific. (B) No viral amplicons were detected in any of the assays. NTC = no template control. Lanes 1 to 15 correspond to the 15 primer sets listed in Table 2, ranging from PAR-F1/PAR-R (1) to Dcon-F/DENV4-R (15). [file TLSR_37-1-293-g00S2.tif]
